# Proposed Amendment to Constitution and By-Laws

**Published:** 1900-08

**Authors:** 


### A. V. M. A.

---

### PROPOSED AMENDMENT TO CONSTITUTION AND BY-LAWS.

WHEREAS, The progress and development of veterinary medicine in America has been slow and arduous; struggling through its infancy without the governmental protection which fosters it in older countries; hampered by the disposition of a large portion of the community toward quackery, home remedies, and patent medicines; impeded by the mercenary motives of many who have sacrificed professional reputation to business success; and

WHEREAS, The growth of our own veterinary association (until within a few years) has been confined to a limited area in the United States, and has remained somewhat conservative and restricted within itself, neither letting the public know its strength nor the profession know its full value; and

WHEREAS, There are still in the United States a number of reputable men and worthy practitioners, who by force of circumstances have not joined our body, but who by their scientific attainments and valuable veterinary services would add value to our association by becoming members;

*Therefore*, We do offer for consideration the following amendments to the constitution and by-laws of the American Veterinary Medical Association:

Chapter VI., Article I. In the second paragraph, after the words "This shall go into effect January 1, 1893," erase the remainder of the paragraph to the words ' 'year 1892" and insert the following: Until and including the executive session of the annual meeting of this Association in 1902, shall be eligible for membership in this Association, non-graduate veterinarians and veterinary graduates of two-year colleges under the following conditions:

1. The applicant shall present with his application blank a certificate signed by the President and Secretary of the State or local veterinary association of the community in which he practices, stating that he is a member in good standing of such association ; or

2. The applicant shall have on his application blank the endorsement of five members of the American Veterinary Medical Association, of whom two at least shall be practitioners of the State of residence of the applicant;

And the provisions of this amendment shall cease at the close of the annual meeting in 1902.

[Signed] RUSH S. HUIDEKOPER,
AUSTIN PETERS,
S. BRENTON.

VETERINARIANS in the vicinity of Philadelphia, Baltimore, and Washington will find the following plan for reaching Detroit convenient and delightful, as well as affording them very favorable rates of travel: On Saturday, September 1st the Reading Railroad will conduct a ten days' excursion to Niagara Falls and return for the sum of ten dollars. Veterinarians taking advantage of this trip could reach Detroit from Niagara Falls or Buffalo by rail or lake at very reasonable rates, or take advantage of the one and one-third rates given from either of these points to Detroit, returning the same way within the time allowed by the ten days' excursion. They could at the same time spend one day (Monday, the 3d) at the Industrial Exposition at Toronto, which will be well worth a visit, going from Toronto direct to Detroit and return to Niagara Falls or Buffalo and continue their homeward journey from that point.

The hot weather of this season suggests that the annual meeting for 1901 should be at the seashore. New Jersey has not had an Association meeting yet. It has within the last year consolidated the three veterinary associations into one—the largest State association in the country. The recent medical congress proved what a delightful place an ocean pier is for a meeting. Atlantic City offers, with all its other amusements, the sandy beach, fishing and bathing in the ocean, which would be a recreation for the families as well as for the members. Let us wash in the surf and start the new century of veterinary medicine at Atlantic City.

Prof. Andrew Smith, of Toronto, President of Canada's great Exposition and Industrial Fair, hopes that the veterinarians will include a visit to Toronto in their route to the annual meeting of the A. V. M. A. at Detroit. From August 27th to September 3d will include a large portion of the exhibition of horses, cattle, etc., and first day of the international dogshow, and much of the racing and contests of high-jumping for hunters.

The Detroit meeting will have much interest added and fresh results of extensive investigations on the subject of "Rabies." In addition to the paper already on the programme by Dr. D. S. White, of Columbus, O., another important contribution on this subject is announced from the busy pen of Dr. D. E. Salmon, chief of the Bureau of Animal Industry, which promises to clear up much of the clouded pathology and nature of this disease.

Dr. Andrew Smith, of Toronto, who, as a Government official of the British Empire, is familiar with the army veterinary service of Great Britain and Canada, has written a note of advice to all the graduates of his school inviting their attention to the importance of aiding in the legislation for the army veterinary bill.

Vice-President Winchester, of Lawrence, Mass., always arranges his summer holiday to include the annual meeting of the A. V. M. A. He will present an interesting paper at Detroit.

Secretary Ray J. Stanclift, of Georgia, has been appointed veterinarian of the Eighth U. S. Cavalry, and after a few days' visit to Buffalo, has gone to Cuba, where he will be stationed.

Dr. Joseph Plaskett, of Nashville, Tenn., and Dr. B. D. Pierce, of Springfield, Mass., are serving as veterinarians with the British troops in South Africa. Dr. George R. White, of Nashville, and Dr. Austin Peters, of Boston, have taken their places as State secretaries.

Two more subjects are announced for the Detroit meeting: "Exercises in Veterinary Dentistry," by Dr. Robert W. Ellis, and "The Clinical Aspect of the Sale-stable Fever of Horses," by Dr. A. H. Baker.

